# The FN1-ITGB4 Axis Drives Acquired Chemoresistance in Bladder Cancer by Activating FAK Signaling

**DOI:** 10.32604/or.2025.072084

**Published:** 2026-01-19

**Authors:** Xiaoyu Zhang, RenFei Zong, Yan Sun, Nan Chen, Kunyao Zhu, Hang Tong, Tinghao Li, Junlong Zhu, Zijia Qin, Linfeng Wu, Aimin Wang, Weiyang He

**Affiliations:** 1 Department of Urology, The First Affiliated Hospital of Chongqing Medical University, Chongqing, 400016, China; 2 Department of Ultrasound, The First Affiliated Hospital of Chongqing Medical University, Chongqing, 400016, China

**Keywords:** Bladder cancer, chemoresistance, fibronectin, focal adhesion kinase, integrin subunit beta 4, gemcitabine and cisplatin

## Abstract

**Objective:**

While cisplatin-based chemotherapy is pivotal for advanced bladder cancer, acquired resistance remains a major obstacle. This study investigates key molecular drivers of this resistance and potential reversal strategies.

**Methods:**

We established GC (Gemcitabine and Cisplatin)-resistant T24-R and UC3-R cell lines from T24 and UM-UC-3 (UC3) cells. Transcriptomic and proteomic analyses identified differentially expressed molecules. Apoptosis and cell viability were assessed by flow cytometry and CCK-8 (Cell Counting Kit-8) assays, while RT-qPCR (Reverse Transcription Quantitative Polymerase Chain Reaction) and Western blot analyzed gene and protein expression. Immunofluorescence evaluated FAK (Focal Adhesion Kinase) phosphorylation, and a xenograft mouse model validated the findings *in vivo*.

**Results:**

Integrated transcriptomic and proteomic analysis identified FN1 (fibronectin) as a consistently upregulated top candidate in resistant cells (T24-R transcript log_2_FC = 2.8, protein log_2_FC = 0.9; UC3-R transcript log_2_FC = 3.7; all *p* < 0.001). Knockdown of FN1 reduced chemoresistance (Resistance Index: 5.2 in T24-R and 2.0 in UC3-R cells, *p* < 0.001) and enhanced apoptosis (approximately 4.5-fold in T24-R and 7.5-fold in UC3-R, *p* < 0.001). ITGB4 (Integrin Subunit Beta 4) was upregulated in resistant cells (transcript log_2_FC: 4.2 in T24-R and 3.03 in UC3-R; protein log_2_FC: 0.67 in T24-R; all *p* < 0.01). Critically, ITGB4 knockdown abolished the chemoresistance promoted by exogenous FN1, which was associated with increased FAK (Y397) phosphorylation.

**Conclusion:**

Our results demonstrate that the FN1-ITGB4 axis drives chemoresistance in bladder cancer via FAK signaling. Targeting this axis represents a promising strategy to overcome chemoresistance.

## 1 Introduction

Globally, bladder cancer ranks among the predominant malignancies affecting the urinary system. The global burden of this disease is significant, accounting for approximately 500,000 new diagnoses and 200,000 fatalities annually, and its occurrence and death toll have been on the rise in recent years [1]. Cisplatin combined with gemcitabine is the first-line regimen for advanced and metastatic bladder cancer [2]. However, the development of subsidiary chemoresistance presents a significant challenge to treatment, often leading to reduced survival times for patients [3]. Therefore, there is a need to actively explore new regimens to counteract chemoresistance, so that the time of benefit to patients can be further prolonged.

FN1 (fibronectin) is a large glycoprotein composed of two smaller subunits and is overexpressed in various malignancies, including gastric and breast cancers [4–7]. FN1 is involved in cell-matrix interactions, including cell invasion and migration, regulation of the cytoskeleton, cell morphology, and differentiation. It binds to various cells, bacteria, and other extracellular matrix proteins, contributing to extracellular matrix formation [8–10]. Mounting evidence now underscores the functional significance of FN1 in mediating resistance to chemotherapy, primarily via the suppression of integrin-mediated apoptotic pathways [11–13]. The integrin family is a bridge between the extracellular matrix and the cytoskeleton, transmitting biochemical and mechanical signals between cells and the environment under various physiological and pathological conditions [14–16]. Integrins likewise play an indispensable role in chemoresistance. They play an important function in chemoresistance by inhibiting apoptosis and other mechanisms in tumors such as breast, lung, and pancreatic cancers [17–19]. Following integrin activation, the Focal Adhesion Kinase (FAK) signaling cascade represents a critical downstream event. This pathway is widely recognized for its pro-survival function and its ability to confer apoptosis resistance.

In this study, we established GC (Gemcitabine and Cisplatin)-resistant bladder cancer cell models. Combining transcriptomic and proteomic screening with rigorous functional validation, we aimed to investigate the role and mechanism of FN1, a top candidate from our screens, and its specific integrin partner ITGB4 in promoting GC resistance, with the ultimate objective of identifying novel therapeutic targets to reverse chemotherapy resistance in bladder urothelial carcinoma. We hypothesized that the FN1-ITGB4 signaling axis promotes GC resistance in bladder cancer by activating FAK-mediated survival signaling and suppressing apoptosis.

## 2 Materials and Methods

### 2.1 Construction of GC-Resistant T24-R and UC3-R Cell Lines

The T24 and UC3 human bladder carcinoma cells were acquired from the Shanghai Institute of Cell Biology, Chinese Academy of Sciences. The specific catalog numbers for T24 and UC3 are TCHu 98 and SCSP-536, respectively. Both cell lines were authenticated by short tandem repeat (STR) profiling upon acquisition and were routinely confirmed to be free of mycoplasma contamination using a PCR (Polymerase Chain Reaction)-based detection kit (Procell, PB180525, Wuhan, China). All experiments were conducted with cells within 20 passages after thawing. The cells were cultured in Dulbecco’s Modified Eagle Medium (Procell, PM150210) supplemented with 10% fetal bovine serum (Procell, 164210) at 37°C in a 5% CO_2_ environment. Notably, no antibiotics (including penicillin-streptomycin) were added to the culture medium throughout the study.

GC (Gemcitabine and Cisplatin) resistance in T24 and UC3 cells was induced by gradually increasing the GC concentration in the culture medium, leading to the establishment of T24-R and UC3-R cell lines. To ensure reproducibility, all subsequent experiments were conducted with cells within a defined passage range (between passages 5 and 20 after the resistant phenotype was stabilized). To establish the GC-resistant sublines (T24-R and UC3-R), parental cells were subjected to intermittent exposure to progressively increasing concentrations of gemcitabine (MedChemExpress, HY-17026, Monmouth Junction, NJ, USA) and cisplatin (MedChemExpress, HY-17394) in T25 flasks containing 5 mL of medium. Each treatment cycle lasted for 72 h, followed by a recovery period in drug-free medium until the cells regained normal proliferation (typically reaching 70%–80% confluence). The total induction process lasted for approximately 1 year. The initial concentrations were 0.1 µg/mL for cisplatin and 0.1 ng/mL for gemcitabine. The drug concentrations were escalated in defined steps (cisplatin: 0.1 → 0.2 → 0.5 → 1.0 → 2.0 µg/mL; gemcitabine: 0.1 → 0.5 → 1 → 5 → 10 ng/mL) once the cells recovered and resumed normal proliferation, typically reaching approximately 70%–80% confluence, after the previous treatment cycle.

The resistance of the resulting T24-R and UC3-R cell lines was confirmed and quantified by determining the half-maximal inhibitory concentration (IC50) using the CCK-8 (Cell Counting Kit-8) (Topscience, C0005, Shanghai, China) assay. For the CCK-8 assay, cells were seeded in 96-well plates at a density of 5 × 10^3^ cells/well and simultaneously exposed to a concentration gradient of gemcitabine (0–10 ng/mL) and cisplatin (0–2 µg/mL). After 48 h of continuous drug treatment, 10 µL of CCK-8 reagent was added to each well, and the plates were incubated at 37°C for 2 h before measuring the absorbance at 450 nm (BioTek, Synergy H1, Winooski, VT, USA). The resistance index (RI) was calculated based on the results using the following formula: RI = IC50_T24-R_/IC50_T24_ (or IC50_UC3-R_/IC50_UC3_).

For exogenous FN1 stimulation, recombinant human FN1 protein (rFN1) (R&D Systems, 1030-FN, Minneapolis, MN, USA) was used. The endotoxin level was confirmed by the manufacturer to be <1.0 EU/μg.

### 2.2 Quantitative Real-Time Polymerase Chain Reaction (qRT-PCR)

Total RNA was extracted using the FastPure Cell/Tissue Total RNA Isolation Kit V2 (Foregene, RE-03113, Beijing, China), and cDNA was synthesized using the ABScript III RT Master Mix for qPCR with gDNA Remover (ABclonal, RK20429, Wuhan, China) according to the manufacturer’s instructions. The primers were designed and synthesized by Tsingke Biotechnology Co., Ltd., Beijing, China. The sequences are listed in Table 1. Human ACTB was used as the internal control. The qPCR reaction was performed using the SsoAdvanced™ Universal SYBR® Green Supermix (Bio-Rad, 1725271, Hercules, CA, USA) on a Bio-Rad CFX96 Real-Time PCR system. The 20 μL reaction volume contained 10 μL of Supermix, 0.8 μL of each forward and reverse primer (10 μM), 2 μL of cDNA template, and 6.4 μL of nuclease-free water. The cycling conditions were as follows: 95°C for 30 s; 40 cycles of 95°C for 10 s and 60°C for 30 s. The relative expression levels of target gene mRNA, normalized to ACTB, were evaluated using the 2^−∆∆Ct^ quantitative method. The primer sequences are provided in Table 1.

**Table 1:** Primer sequences used for RT–qPCR

Gene name	Forward sequence (5^′^-3^′^)	Reverse sequence (5^′^-3^′^)
FN1	GAGGGCAGAAGAGACAA CATGAA	CCCTTCATTGGTTGTGCA GATTT
ACTB	CATGTACGTTGCTATC CAGGC	CTCCTTAATGTCACG CACGAT

### 2.3 Plasmids and siRNA

All specific small interfering RNAs (siRNAs) and plasmids for gene knockdown were sourced from Tsingke Biotechnology Co., Ltd. with the corresponding oligonucleotide sequences detailed in Table 2. For transfection, cells in the logarithmic growth phase were seeded in 12-well plates at a density of 1 × 10^5^ cells per well and cultured for 24 h to reach 60%–70% confluence. According to the manufacturer’s instructions, Lipofectamine 2000 (Invitrogen, 11668030, Carlsbad, CA, USA) was used to transfect plasmids/siRNAs into T24-R and UC3-R cell lines. Specifically, for each well, 2.5 μg of plasmid or 100 pmol of siRNA was diluted in 125 μL of Opti-MEM Reduced-Serum Medium (Gibco, 31985070, Waltham, MA, USA), which was then mixed with a solution containing 5 μL of Lipofectamine 2000 reagent diluted in 125 μL of Opti-MEM Reduced-Serum Medium. After a 20-min incubation at room temperature, the mixture was added dropwise to the cells. Subsequent functional experiments were conducted 48 h after transfection.

**Table 2:** The sequences of shRNAs/siRNAs used in the study

shRNA/siRNA	Sequence (5^′^-3^′^)
NC	TGTTCTCCGAACGTGTCACGTTTCAAGAGAACGTGA CACGTTCGGAGAACTTTTTTC
FN1-1	GGAGUUGAUUAUACCAUCA
FN1-2	GUCCUGUCGAAGUAUUUAU
ITGB4	GTGGATGAGTTCCGGAATAAA

### 2.4 Lentivirus Transfection

Stable cell lines with knockdown of FN1 and ITGB4 (shFN1 and shITGB4), along with a negative control (shNC), were established using a lentiviral-mediated approach. The lentiviral vectors and associated short hairpin RNA (shRNA) constructs were procured from Tsingke Biotechnology Co., Ltd. (Beijing, China), and the specific targeting sequences are provided in Table 2. When T24-R or UC3-R cells attained approximately 50% confluency, they were subjected to lentiviral transduction with a multiplicity of infection (MOI) set at 20. Polybrene (Sigma-Aldrich, TR-1003, St. Louis, MO, USA) at a final concentration of 8 μg/mL was utilized to enhance the infection efficiency, and the transfection was conducted at 37°C for 24 h. After transduction, the supernatant was replaced with DMEM supplemented with 10% FBS, and the cells were further cultured at 37°C in a 5% CO_2_ atmosphere for 48 h. Subsequently, puromycin (2 µg/mL) was added to the medium to promote the stable selection of transduced cell lines. All the sequences of shRNA and siRNAs were listed in Table 2.

### 2.5 Cell Proliferation Assay

Cell proliferation was assessed using the CCK-8 assay. Briefly, cells were seeded into 96-well plates at a density of 5 × 10^3^ cells per well and treated with a gradient of cisplatin intervention concentrations (0, 5, 10, 15, 20, 25 µg/mL) and a gradient of gemcitabine intervention concentrations (0, 2.5, 5, 10, 100, 200 ng/mL). After incubation for 48 h, the cells were incubated with 10 μL of CCK-8 reagent for 1 h in the dark. Absorbance was measured at 450 nm using a microplate reader (BioTek, Synergy H1, Winooski, VT, USA) and used for the calculation of cell viability.

### 2.6 Western Blot

The total protein was extracted using RIPA Lysis Buffer (Meilunbio, MA0151, Dalian, China). To validate FN1 knockdown efficiency under normal culture conditions (as shown in Supplementary Fig. S2), protein lysates were prepared from T24-R and UC3-R cells transfected with FN1-targeting or control shRNA after 48 h of transfection without drug treatment. The protein concentration was determined using a BCA Protein Assay Kit (Beyotime, P0012, Shanghai, China). Sodium dodecyl sulfate polyacrylamide gel electrophoresis (SDS-PAGE) was chosen for the total protein separation. Each lane received an equal protein load of 20 *μ*g. The proteins were then transferred to nitrocellulose membranes (the membranes were cut horizontally). The membranes were blocked with 5% non-fat milk in TBST at room temperature for 1 h. The membranes were incubated with primary antibodies at 4°C overnight, including anti-FN1 (1:1000; HUABIO, ET1702-25, Hangzhou, China), anti-ITGB4 (1:1000; HUABIO, ET1703-52), anti-Bcl-2 (1:1000; HUABIO, ET1702-53), anti-BAX (1:1000; HUABIO, ET1603-34), anti-Caspase 3 (1:1000; Cell Signaling Technology, 9664, Danvers, MA, USA), anti-FAK (1:5000; HUABIO, ET1602-25), anti-p-FAK (Y397) (1:1000; HUABIO, ET1610-34), and anti-ACTB (1:10,000; Proteintech, 66009-1-Ig, Rosemont, IL, USA). Following primary antibody incubation, the membranes were incubated with species-matched horseradish peroxidase (HRP)-conjugated secondary antibodies at room temperature for 1 h. The secondary antibodies used were: goat anti-rabbit IgG (1:5000; Abbkine, A21020, Wuhan, China) and goat anti-mouse IgG (1:5000; Abbkine, A21010, Wuhan, China). After secondary antibody incubation, the membranes were washed three times with TBST buffer (5 min per wash) to remove unbound secondary antibodies. Subsequently, enhanced chemiluminescence (ECL) detection reagent (Meilunbio, MA0186, Dalian, China) was added dropwise to the membrane surface and incubated at room temperature in the dark for 5 min. Finally, the protein bands were visualized using a chemiluminescence imaging system (Tanon, 5200, Shanghai, China).

### 2.7 Cell Immunofluorescence

Different groups of cells were fixed using 4% paraformaldehyde for 30 min (RT), followed by three gentle washes with PBS. Subsequently, they were treated with 0.3% Triton X-100 for 15 min and washed three times gently with PBS. The cells were then incubated with 200 μL of 10% goat serum (Beyotime, C0265) at room temperature for 30 min. After removing the goat serum, the cells were incubated overnight at 4°C with the anti-p-FAK (Y397) antibody (1:200; HUABIO, ET1610-34). After three gentle washes with PBS, a diluted secondary antibody (1:500; Beyotime, A0423), Alexa 488-conjugated goat anti-rabbit IgG, was added and incubated at room temperature for 1 h. After counterstaining with DAPI (Beyotime, C1002, Shanghai, China) for 5–10 min at room temperature in the dark, the cells were observed under a confocal microscope (Zeiss, LSM 900, Oberkochen, Germany).

### 2.8 Co-Immunoprecipitation (Co-IP)

Co-IP assays were conducted to confirm the association of FN1 with ITGB4. First, T24-R cells (seeded at a density of 2 × 10^6^ cells per 10-cm dish) at over 80% confluency were washed 2–3 times with pre-cooled PBS. After removing the PBS, 500 μL of IP buffer containing 5 μL of PMSF was added, and the cells were gently shaken on a 4°C shaker for 30 min. Subsequently, the cells were scraped off using a cell scraper, sonicated to disrupt the cell walls, and centrifuged at 12,000× *g* for 15 min at 4°C. The supernatant was then transferred, and both IP and IgG control groups were set up. The IP buffer was composed of 20 mM Tris-HCl (pH 7.5), 150 mM NaCl, 1% NP-40, 1 mM EDTA, and a protease inhibitor cocktail. The IgG group was treated with 2.5 μg of IgG antibody, while the IP group was treated with 2.5 μg of the target antibody, including anti-FN1 (Abcam, EPR19241-46, Cambridge, UK) and anti-ITGB4 (Proteintech, 21738-1-AP, Rosemont, IL, USA), and both were incubated overnight at 4°C. The next day, 30 μL of magnetic beads were added to each group, and the beads were washed 3–4 times with PBS containing 2% Tween-20 using a magnetic rack. The liquid from both the IgG and IP groups was added to EP tubes containing the magnetic protein A/G beads (Thermo Fisher Scientific, 26162, Waltham, MA, USA) and incubated for 6–8 h. After incubation, the EP tubes were placed on the magnetic rack for 1 min, and the liquid was discarded. The beads were washed with 1 mL of IP buffer, placed on the magnetic rack for 1 min, and the buffer was discarded. This washing step was repeated 3–4 times. Finally, 30 μL of protein loading buffer was added, and the samples were boiled at 100°C for 8 min before being used for subsequent Western blot analysis for validation.

### 2.9 Flow Cytometry

Flow cytometric analysis was performed using a BD Accuri C6 flow cytometer (BD Biosciences, San Jose, CA, USA). Apoptosis was detected using the FITC Annexin V Apoptosis Detection Kit II (BD Biosciences, 556547, San Jose, CA, USA). Briefly, cells were harvested and resuspended at a density of 1 × 10^6^ cells/mL. According to the manufacturer’s protocol, the cell suspension was simultaneously stained with Annexin V-FITC and propidium iodide (PI) in the dark at room temperature for 15 min. The apoptosis rate was calculated as the percentage of early and late apoptotic cells. The data were analyzed using FlowJo software (Version 10.8.1; BD Life Sciences, Ashland, OR, USA).

### 2.10 Enzyme-Linked Immunosorbent Assay

T24-R or UC3-R cells (seeded at 5 × 10^5^ cells per well in 6-well plates) were incubated in 5 mL of culture medium for 48 h. Following collection, the supernatant was subjected to centrifugation (1500× *g*, 10 min, 4°C) to remove any dislodged cells. The resulting sample was subsequently analyzed using the Human FN1 Quantikine ELISA Kit (R&D Systems, DY1918, Minneapolis, MN, USA). All ELISA procedures strictly followed the supplier’s protocol. Absorbance readings were obtained at 450 nm, with 570 nm as the reference wavelength, employing a microplate reader (BioTek, Synergy H1, Winooski, VT, USA).

### 2.11 Immunohistochemistry (IHC)

The primary tumor specimens in this study were obtained through radical cystectomy from bladder cancer patients who were diagnosed and received neoadjuvant chemotherapy at the First Affiliated Hospital of Chongqing Medical University. The neoadjuvant chemotherapy regimen in this study consisted of gemcitabine (1000–1200 mg/m^2^, administered as a 30-min intravenous infusion on days 1 and 8) combined with cisplatin (70 mg/m^2^, administered as a 6–8-h intravenous infusion on day 1), with each cycle lasting 21 days. A total of 4 cycles were typically recommended. Based on tumor response to neoadjuvant chemotherapy, patients were categorized into chemotherapy-sensitive and chemotherapy-resistant groups. The chemotherapy-sensitive group was defined as patients who achieved significant tumor shrinkage or complete response during initial treatment, while the chemotherapy-resistant group comprised patients who initially responded but subsequently relapsed and developed drug resistance. The assessment of response to neoadjuvant chemotherapy was based on histopathological findings from cystectomy specimens obtained after completion of neoadjuvant chemotherapy. Follow-up information was collected by reviewing the patients’ medical records. This study was approved by the Ethics Committee of the First Affiliated Hospital of Chongqing Medical University. A total of 12 patient specimens (6 from the chemotherapy-sensitive group and 6 from the chemotherapy-resistant group) with pathologically confirmed bladder urothelial carcinoma were included in the IHC analysis. The study involving human specimens was approved by the Ethics Committee of the First Affiliated Hospital of Chongqing Medical University (Approval No.: 2023444). All procedures were performed in accordance with the ethical standards of this committee and with the informed consent obtained from all individual participants. All methods were carried out in accordance with the relevant guidelines and regulations, including the Declaration of Helsinki.

Paraffin-embedded sections of clinical bladder cancer tissues were deparaffinized in xylene, rehydrated in graded ethanol, and microwaved in 10 mM sodium citrate (pH 6.0) for 15 min. Hydrogen peroxide (0.3%) was used to block endogenous peroxidase activity. Following blocking with 10% normal goat serum, tissue sections were probed with a primary antibody against FN1 (HUABIO, ET1702-25), diluted at 1:200, in a 4°C overnight incubation. Subsequently, a horseradish peroxidase (HRP)-conjugated goat anti-rabbit IgG secondary antibody (1:500; ZSGB-BIO, PV-6001, Beijing, China) was applied and allowed to incubate for 1 h at room temperature. The sections were visualized using a DAB detection kit (Maixin Bio, K026, Fuzhou, China). The sections were counterstained with hematoxylin. The stained sections were observed and imaged under a light microscope (Nikon, Eclipse E100, Tokyo, Japan). The images were analyzed using ImageJ software (Version 1.53t; National Institutes of Health, Bethesda, MD, USA).

### 2.12 In Vivo Tumor Xenograft Study

Twenty male BALB/c nude mice (4–5 weeks old, 16–18 g body weight) were acquired from Vital River Laboratory (Beijing, China) and subsequently allocated at random into four experimental cohorts (5 mice per group), designated as follows: shNC + Saline, shNC + Cisplatin, shFN1 + Saline, and shFN1 + Cisplatin. The group allocation was concealed from the personnel performing the drug administration and tumor measurement. Mice were housed under specific pathogen-free (SPF) conditions with a 12/12-h light/dark cycle, provided with standard laboratory diet and water ad libitum, and acclimatized for one week prior to experiments. Lentivirus-transduced T24-R cells (5 × 10^6^) in 100 μL of PBS were subcutaneously injected into the axillary region of the mice. One week post-inoculation, mice in the cisplatin-treated groups received an intraperitoneal injection of cisplatin (2.5 mg/kg) every 3 days, while the control groups received an equal volume of 0.9% saline (200 μL per injection). Tumor volume and body weight were measured every 3 days. Tumor volume was calculated using the formula: V = (Length × Width^2^)/2. The experimental endpoint was established at 24 days following inoculation. At this predetermined time, all mice underwent euthanasia by CO_2_ asphyxiation; subsequently, the tumors were excised and their mass was recorded. No. subjects were omitted from the final dataset. The animal study protocol was approved by the Institutional Animal Care and Use Committee (IACUC) of Chongqing Medical University (IACUC-CQMU-2024-0893).

### 2.13 TUNEL Staining

For tissue section preparation, tumor samples were fixed in 4% paraformaldehyde, embedded in paraffin, and sectioned at a thickness of 4 μm. Following deparaffinization in xylene and rehydration through a graded ethanol series (100%, 95%, 85%, 75%), antigen retrieval was performed using citrate buffer (pH 6.0) at 95°C for 15 min. Apoptotic cells within tumor tissues were identified by terminal deoxynucleotidyl transferase dUTP nick-end labeling (TUNEL) staining, which was carried out following the supplier’s protocol using a one-step TUNEL *in situ* apoptosis detection kit (Green, Elab Fluor® 488, Elabscience, E-CK-A321, Wuhan, China). Sections were permeabilized with 0.1% Triton X-100 in PBS for 20 min at room temperature prior to TUNEL reaction. Following TUNEL staining, the samples were subjected to nuclear counterstaining utilizing DAPI (Beyotime, C1002) for a duration of 5 min. Fluorescence imaging was then performed with a microscope (Nikon, Eclipse Ci-L, Tokyo, Japan), maintaining identical exposure conditions for every sample. For image analysis, five random fields (200× magnification) were captured per section. Quantification of TUNEL-positive (green) cells and the total count of DAPI-stained (blue) nuclei per field of view was conducted automatically. This was achieved through the “Analyze Particles” module in ImageJ (Version 1.53t; National Institutes of Health), with uniform threshold parameters employed for all micrographs. The apoptosis index was calculated as: (TUNEL-positive cells/total DAPI-positive nuclei) × 100%.

### 2.14 Transcriptomic and Proteomic Analyses

For transcriptomic analysis, total RNA was extracted from T24/T24-R and UC3/UC3-R cells using TRIzol reagent (Invitrogen, 15596026, Carlsbad, CA, USA). RNA purity and integrity were assessed using a NanoDrop spectrophotometer (Thermo Scientific, Wilmington, DE, USA) and an Agilent 2100 Bioanalyzer (Agilent Technologies, Santa Clara, CA, USA), respectively, with all samples having an A260/A280 ratio between 1.8–2.0 and an RNA Integrity Number (RIN) >7.0. RNA-seq libraries were prepared using the NEBNext® Ultra™ RNA Library Prep Kit (NEB, E7530, Ipswich, MA, USA) and sequenced on an Illumina NovaSeq 6000 platform with 150 bp paired-end reads. Quality control of the raw sequencing reads was conducted with Fastp v0.23.2, followed by alignment against the human reference genome GRCh38 via HISAT2 v2.2.1. Subsequent steps involved gene expression quantification implemented by featureCounts v2.0.1, and differential expression analysis executed using DESeq2 v1.38.3. Genes with |log2(Fold Change)| > 1 and an adjusted *p*-value (FDR) < 0.05 were considered significantly differentially expressed.

For proteomic analysis, total protein was extracted from T24/T24-R cells using RIPA lysis buffer (Beyotime, P0013B) containing protease inhibitors. The protein concentration was determined using a BCA assay kit (Beyotime, P0012). Protein samples were digested with trypsin (Sequencing Grade Modified Trypsin, Promega, V5111, Madison, WI, USA) at a 1:50 enzyme-to-substrate ratio for 16 h at 37°C. The resulting peptides were separated using an EASY-nLC 1200 system (Thermo Scientific, Waltham, MA, USA) with a C18 analytical column (Thermo Scientific, ES903, 75 μm × 250 mm, 2 μm particle size). The LC separation used a 120-min gradient from 5% to 35% mobile phase B (0.1% formic acid in 80% acetonitrile) at a flow rate of 300 nL/min. Mass spectrometry analysis was performed on an Orbitrap Exploris™ 480 mass spectrometer (Thermo Scientific, Waltham, MA, USA) in data-dependent acquisition (DDA) mode. The raw data were processed using MaxQuant v2.1.3.0 software against the UniProt Human database (release 2023_01) with the following parameters: precursor mass tolerance of 10 ppm, fragment mass tolerance of 0.02 Da, fixed modification of carbamidomethylation (C), variable modifications of oxidation (M) and acetylation (protein N-term), maximum of two missed cleavages, and a false discovery rate (FDR) of 1% at both peptide and protein levels. Proteins with |log2(Fold Change)| > 1 and an adjusted *p*-value (FDR) < 0.05 were considered significantly altered.

### 2.15 Integrated Omics Analysis and Visualization

**Figure 1:**
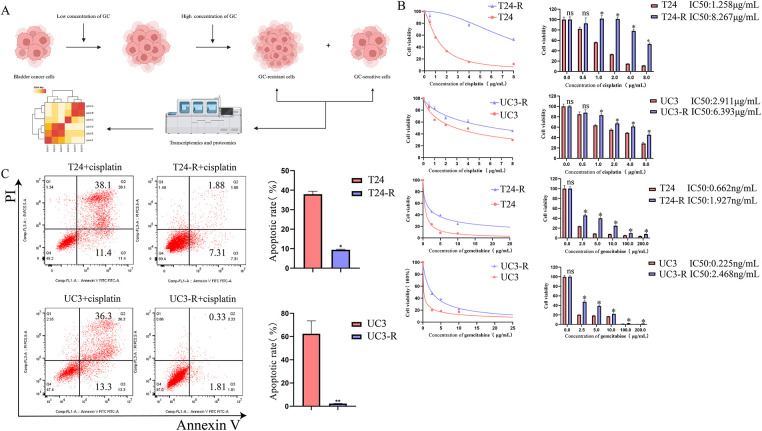

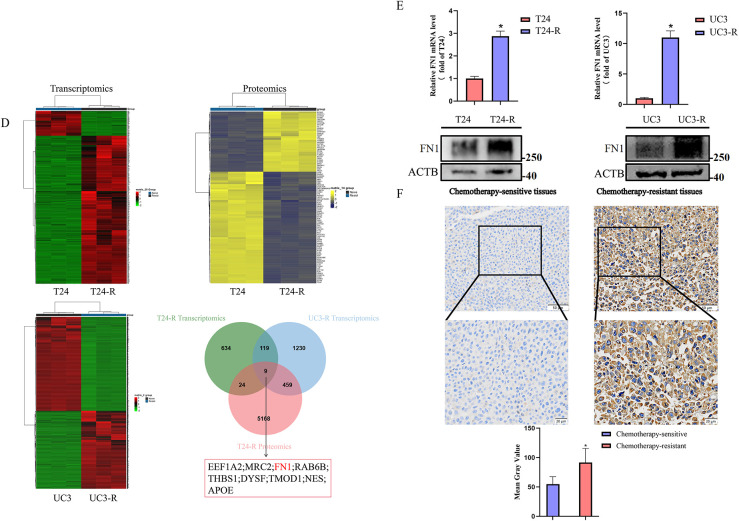
Establishment and characterization of GC (Gemcitabine and Cisplatin)-resistant bladder cancer cell lines and identification of resistance-related proteins. (**A**) GC-resistant T24-R and UC3-R cell lines were generated by gradually increasing GC concentrations. Created in BioRender (https://BioRender.com). (**B**) Dose-response curves and calculated half-maximal inhibitory concentration (IC50) values for cisplatin (upper panel) and gemcitabine (lower panel) in parental (T24, UC3) and GC-resistant (T24-R, UC3-R) cell lines. Data are presented as the mean ± SD from at least three independent experiments. (**C**) Apoptosis rates of parental and resistant cell lines after treatment with cisplatin, as determined by flow cytometry. Data are presented as the mean ± SD (*n* ≥ 3). (**D**) Transcriptomic and proteomic analyses identified FN1 (Fibronectin), EEF1A2 (Eukaryotic Translation Elongation Factor 1 Alpha 2), MRC2 (Mannose Receptor C-Type 2), RAB6B (RAB6B, Member RAS Oncogene Family), THBS1 (Thrombospondin 1), DYSF (Dysferlin), TMOD1 (Tropomodulin 1), NES (Nestin), and APOE (Apolipoprotein E) as overexpressed in GC-resistant cells. (**E**) RT-qPCR (Reverse Transcription Quantitative Polymerase Chain Reaction) and Western blot confirmed FN1 overexpression in T24-R and UC3-R. (**F**) FN1 staining was stronger in GC-resistant bladder cancer tissues (Chemotherapy Sensitive Group: *n* = 6; Chemotherapy Resistant Group: *n* = 6). For all panels, a *t*-test was used for comparisons between two groups, and one-way ANOVA was used for comparisons among multiple groups. Statistical significance was set at *p* < 0.05. ns, not significant, *p < 0.05, **p < 0.01

The integration of transcriptomic (GSE309386, GSE309388 from GEO) and proteomic (PXD069140 from ProteomeXchange) datasets was performed to identify consistently dysregulated molecules. Significantly altered genes and proteins were defined as those with an absolute log_2_ fold change (|log_2_FC|) > 1 and an adjusted *p*-value < 0.05. The overlap of significantly altered genes and proteins was visualized using a Venn diagram (Fig. 1D). The concordant upregulation of FN1, as displayed in Fig. S1, was illustrated using a scatter plot generated with the ggplot2 package (version 3.4.2) in R software (version 4.2.1).

### 2.16 Molecular Docking

The molecular docking study was performed to predict the interaction between FN1 and ITGB4. The three-dimensional structure of the FN1 protein (P02751) was retrieved from the AlphaFold Database (AF-P02751-F1). The structure of the ITGB4 subunit was obtained from the UniProt database (P16144) and modeled using the AlphaFold2 algorithm. Molecular docking was carried out using the HDOCK server (http://hdock.phys.hust.edu.cn/). The docking parameters were set to default, and the results were visualized based on the highest confidence score. The binding pose with the best docking score and highest confidence was selected for further analysis and visualization (using PyMOL, Version 2.5.4, Schrödinger, LLC.) in Fig. 5B.

### 2.17 Statistical Analysis

All experiments were independently repeated at least three times. The value ‘n’ refers to the number of independent biological replicates. Statistical analyses were conducted employing GraphPad Prism 8.0 (GraphPad Software Inc., San Diego, CA, USA). Continuous data are expressed as the mean ± standard deviation (SD), and the normality of distribution was verified by the Shapiro-Wilk test. A two-tailed *t*-test was performed to compare the differences between two groups. Intergroup differences were evaluated by one-way ANOVA, with Tukey’s test utilized for subsequent pairwise comparisons. A *p*-value of less than 0.05 was considered statistically significant.

## 3 Results

### 3.1 FN1 Is Overexpressed in GC-Resistant Bladder Cancer Cell Lines and Tissues

GC-resistant bladder cancer cell lines were screened using increasing concentrations of cisplatin and gemcitabine, and when the cell lines developed significant GC resistance, transcriptomics and proteomics were performed to screen for key genes for resistance (Fig. 1A). The resistance of the cell lines was determined by calculating the half-maximal lethal concentration (IC50) of cisplatin and using flow cytometry. Ultimately, GC-resistant cell lines T24-R and UC3-R were successfully established. The results showed that the IC50 of the resistant strain was significantly higher than that of the parental strain (e.g., the IC50 for cisplatin increased 6.57-fold in T24-R cells, *p* < 0.001; Fig. 1B). The resistance indices (RI) for cisplatin in T24-R and UC3-R were 6.57 and 2.2, respectively, and for gemcitabine were 2.91 and 10.97, respectively, indicating a significant acquisition of drug resistance. Fig. 1B illustrates the dose-response curves for cisplatin (upper panel) and gemcitabine (lower panel). A substantial rise in the IC50 values of both chemotherapeutic agents was observed in the resistant cell models. The apoptosis rate of the cisplatin-treated resistant strain was significantly lower than that of the parental strain (Fig. 1C). By cross-referencing the analysis results using Venn diagrams, we found that FN1, EEF1A2, MRC2, RAB6B, THBS1, DYSF, TMOD1, NES, and APOE were overexpressed in the resistant cell lines (Fig. 1D). Among these candidates, FN1 was selected for further investigation due to its consistent and marked overexpression at both the transcriptomic and proteomic levels (Fig. S1), its well-characterized role as an ECM ligand for integrins, and its established links to chemoresistance in other cancer types. We chose to focus subsequent mechanistic studies on overcoming resistance to cisplatin, the core DNA-damaging component of the GC regimen. This approach simplifies the experimental system by focusing on a unified cell death pathway, thereby enabling a clearer dissection of the specific signaling mechanisms involved. RT-qPCR and Western blotting analyses confirmed that FN1 was overexpressed in T24-R and UC3-R (Fig. 1E). Similarly, overexpression of FN1 was observed in GC-resistant bladder cancer tissues (Fig. 1F).

### 3.2 FN1 Mediates Chemotherapy Resistance by Inhibiting Apoptosis in Bladder Cancer Cells

**Figure 2:**
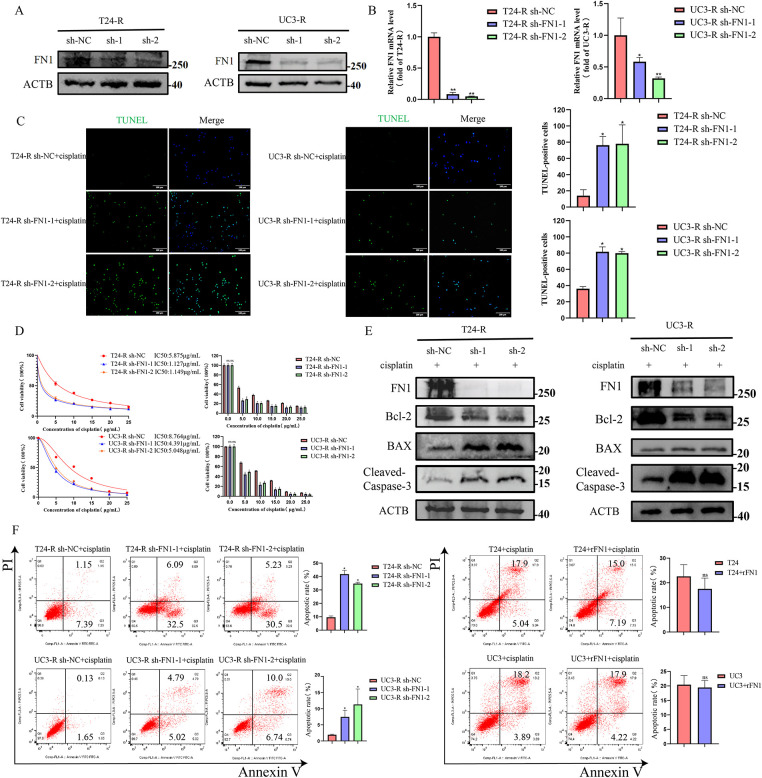
FN1 silencing enhances cisplatin-induced apoptosis in bladder cancer cells. (**A**) FN1 knockdown efficiency in T24-R and UC3-R cells was confirmed by Western blot analysis. (**B**) FN1 knockdown efficiency in T24-R and UC3-R cells was confirmed by RT-qPCR analysis. (**C**) Silencing FN1 significantly increased apoptosis in the resistant cell lines by TUNEL staining. (**D**) Silencing FN1 reduced the IC50 of cisplatin in the resistant cell lines by CCK-8 assay. (**E**) In resistant cells, FN1 knockdown induced the expression of pro-apoptotic mediators, including BAX and cleaved-caspase-3, but suppressed levels of the anti-apoptotic protein Bcl-2. (**F**) Cell apoptosis rates were quantified by flow cytometry. For all panels, a t-test was used for comparisons between two groups, and one-way ANOVA was used for comparisons among multiple groups. Statistical significance was set at *p* < 0.05. ns, not significant, *p < 0.05, **p < 0.01

**Figure 3:**
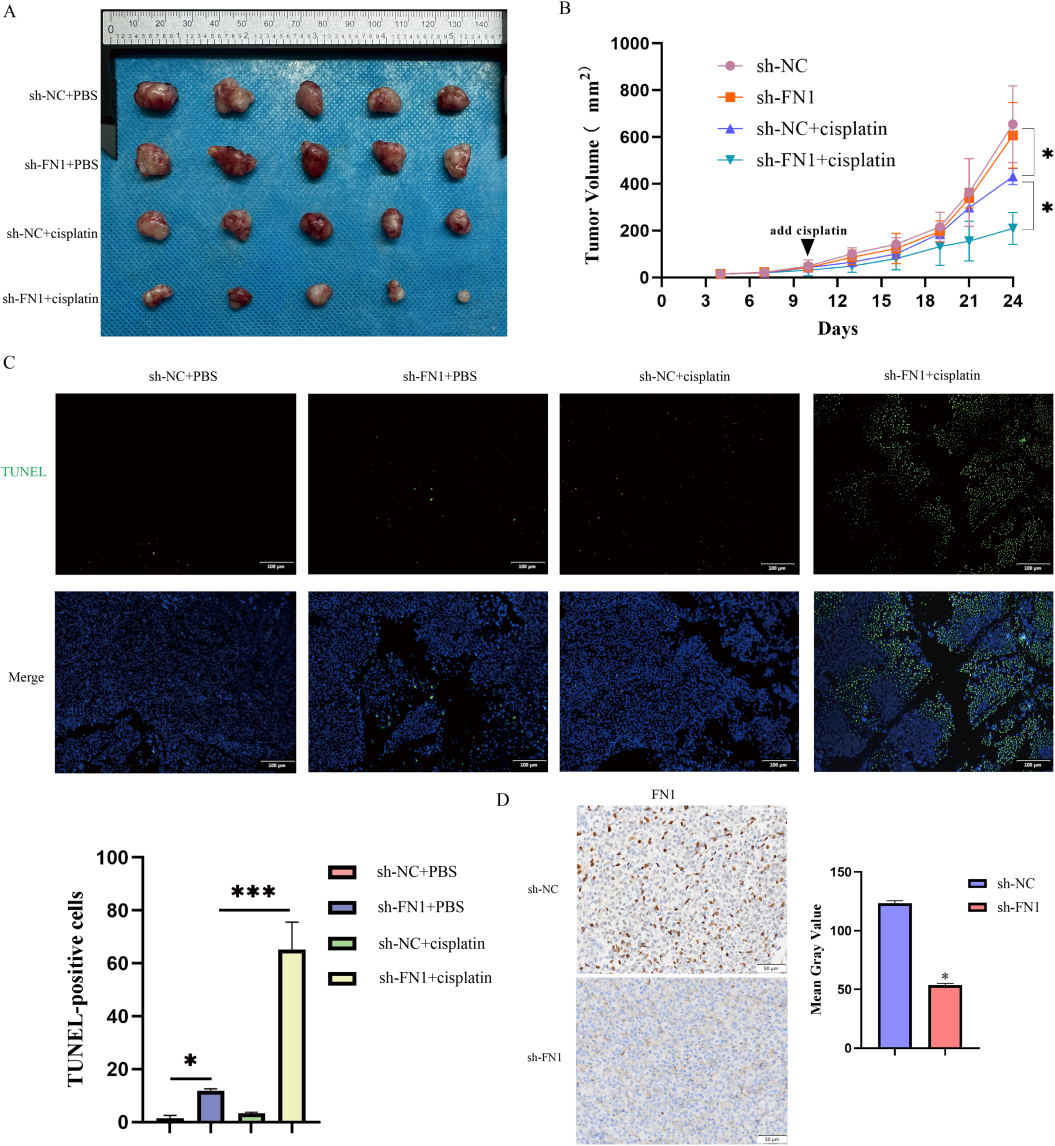
FN1 silencing inhibits tumor growth *in vivo*, enhancing cisplatin sensitivity. (**A**) Representative images of tumors from each treatment group. (**B**) Tumor volume growth curves over time measured in the T24-R xenograft model. (**C**) Apoptosis in tumor tissues was detected by TUNEL staining. (**D**) Immunohistochemical analysis showed decreased FN1 expression in tumor sections from the FN1 knockdown group. For all panels, a *t*-test was used for comparisons between two groups, and one-way ANOVA was used for comparisons among multiple groups. Statistical significance was set at *p* < 0.05. *p < 0.05, ***p < 0.001

Inhibition of apoptosis is one of the key mechanisms of chemotherapy resistance [20]. We silenced FN1 in T24-R and UC3-R cells and assessed the knockdown efficiency using RT-qPCR and Western blot analyses (Fig. 2A,B). We observed that treatment with cisplatin after silencing FN1 in drug-resistant cell lines resulted in a significant increase in the number of apoptotic cells and a marked decrease in IC50 (Fig. 2C,D,F). Based on preliminary experiments, it has been determined that knocking down FN1 in drug-resistant cell lines does not affect apoptosis in the absence of cisplatin (Fig. S2). Therefore, all subsequent experiments were conducted under the condition of cisplatin intervention. Silencing FN1 mediated the increase in apoptosis-related proteins BAX and cleaved-caspase-3, and the decrease in Bcl-2 in the resistant cell lines (Fig. 2E). A xenograft model was generated by implanting either control or FN1-knockdown T24-R cells into the axillary area of BALB/c nude mice. This model was employed to assess how FN1 silencing influences tumor progression and the efficacy of cisplatin under *in vivo* conditions. One week later, an equal amount of cisplatin (2.5 mg/kg) or 0.9% saline was injected intraperitoneally every three days. Notably, in the cisplatin-treated mice, the expansion of xenograft tumors was significantly inhibited, as evidenced by a reduction in tumor volume and an increase in the apoptosis rate of tumor cells. Combining FN1 knockdown with cisplatin treatment resulted in an even more pronounced reduction in tumor volume and a further increase in the apoptosis rate of tumor cells (Fig. 3A–C). Immunohistochemistry showed that FN1 expression was significantly reduced in tumor sections from the FN1 knockdown group (Fig. 3D). These results indicate that silencing FN1 enhances the pro-apoptotic effect of cisplatin on tumor cells, thereby inhibiting resistance.

### 3.3 FN1 Mediates Chemotherapy Resistance by Activating the Phosphorylation of FAK (Y397)

**Figure 4:**
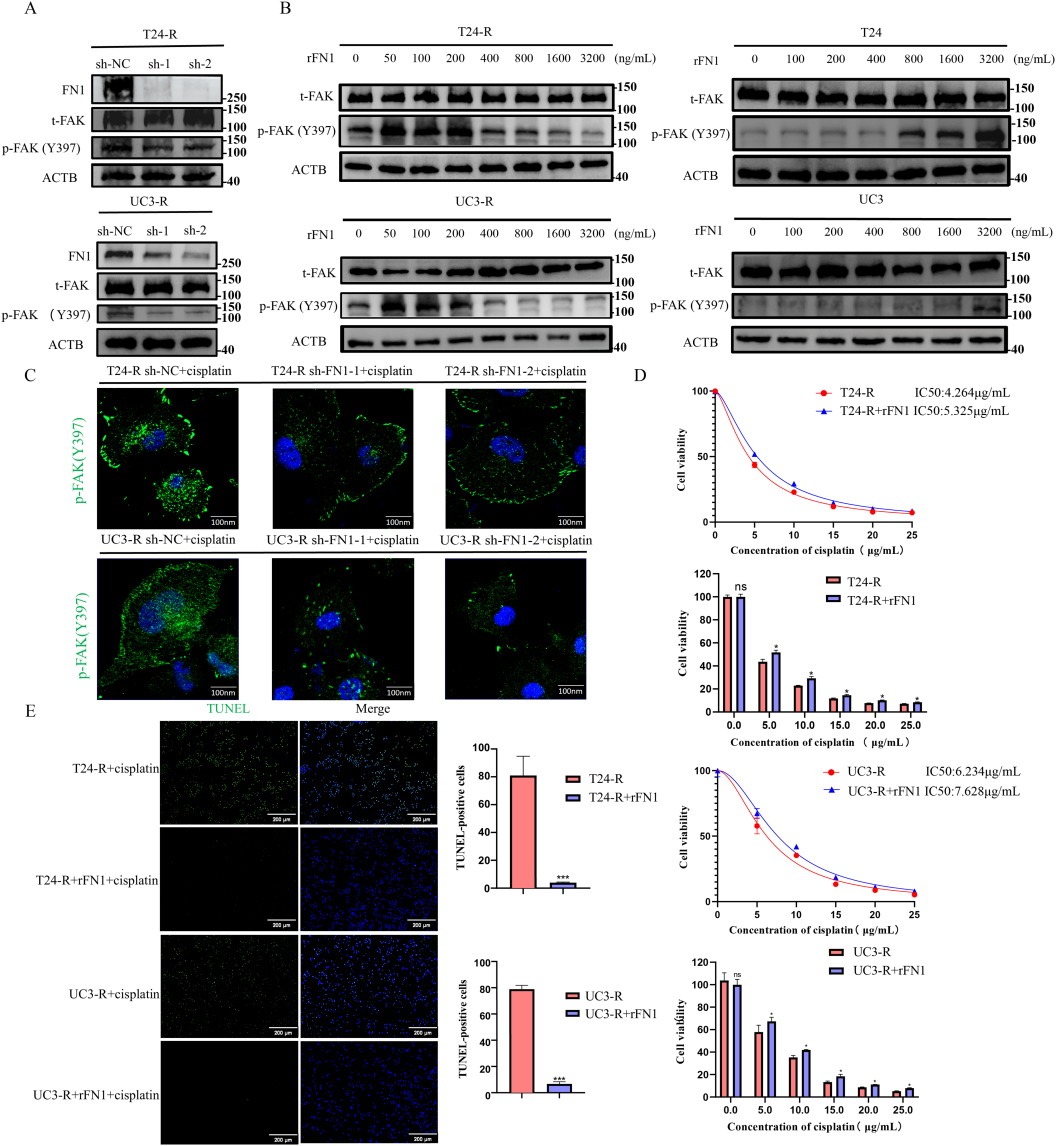
FN1 regulates FAK (Y397) phosphorylation and mediates cisplatin resistance in bladder cancer cells. (**A**) Silencing FN1 in resistant cell lines reduced the phosphorylation of FAK (Y397) as detected by Western blot. (**B**) T24 and UC3 parental and resistant cells were treated with a fixed dose of cisplatin along with a gradient of rFN1 for 48 h. Phosphorylation of FAK (Y397) was assessed by Western blot. Resistant cells (T24-R, UC3-R) showed sensitivity to rFN1 at lower concentrations under cisplatin stress. (**C**) Silencing FN1 in resistant cell lines reduced the phosphorylation of FAK (Y397) as detected by immunofluorescence. (**D**) rFN1 addition increased resistance index in resistant strains. (**E**) The addition of rFN1 reduced apoptosis in resistant strains. For all panels, a *t*-test was used for comparisons between two groups, and one-way ANOVA was used for comparisons among multiple groups. Statistical significance was set at *p* < 0.05. ns, not significant, *p < 0.05, ***p < 0.001

FN1, as a secretory protein, often exerts its biological functions by binding to integrin family receptors and activating the intracellular downstream signaling mediator focal adhesion kinase (FAK) [21]. Silencing FN1 in the resistant strains was found to reduce the phosphorylation levels of FAK (Y397) (Fig. 4A,C). Since FN1 typically functions as a secretory protein, we measured FN1 levels in the supernatants of resistant and parental strains. The results showed that FN1 levels were significantly higher in the resistant strains (Fig. S3). The presence of elevated secreted FN1 is consistent with an autocrine mechanism; therefore, we used rFN1 for subsequent experiments. To directly test whether FN1 can activate pro-survival signaling under chemotherapeutic stress, we examined the effect of rFN1 on FAK phosphorylation in cells simultaneously challenged with cisplatin. Consistent with previous reports [22], our preliminary titration experiment (Fig. 4B) indicated that excessive concentrations of rFN1 could lead to a suboptimal activation response of the downstream pathway. Therefore, identifying an optimal concentration for intervention was crucial. Interestingly, when parental cells (T24 and UC3) were co-treated with cisplatin and rFN1, relatively high concentrations of rFN1 were required to induce significant phosphorylation of FAK (Y397). In contrast, resistant cell lines exhibited noticeable FAK (Y397) phosphorylation at much lower rFN1 concentrations under the same cisplatin stress conditions (Fig. 4B). Therefore, in subsequent experiments, we chose 100 ng/mL rFN1 for intervention. Focal adhesion kinase (FAK) serves as a critical constituent of focal adhesions. The subcellular distribution of FAK is predominantly controlled by integrin binding to extracellular matrix (ECM) components. When integrins bind to the ECM (such as fibronectin, collagen, etc.), FAK is recruited to the focal adhesion region [23,24]. Within focal adhesions, FAK enhances its stability and function through autophosphorylation (e.g., phosphorylation at tyrosine 397) [25,26]. Although under cellular stress or specific signaling stimuli, FAK may enter the nucleus via a nuclear localization signal (NLS) to participate in functions such as DNA repair or transcriptional regulation [27], in this experiment, FN1 regulates FAK phosphorylation levels through an autocrine mechanism, thus FAK is primarily localized to the focal adhesion regions on the cell membrane. Experimental findings revealed that the exogenous addition of rFN1 enhanced the phosphorylation of FAK (Y397) in the resistant strains, leading to an increased resistance index (Figs. 4B,D and S4). The addition of rFN1 in the resistant strains reduced the number of apoptotic cells (Figs. 2F and 4E). Collectively, these findings demonstrate a regulatory role for FN1 expression in modulating FAK (Y397) phosphorylation, thereby influencing chemoresistance. In contrast, FN1 exhibited minimal impact on FAK (Y397) phosphorylation levels within the parental cell lines.

### 3.4 FN1 Mediates the Regulation of FAK (Y397) Phosphorylation in an ITGB4-Dependent Manner, Thereby Inhibiting Apoptosis

**Figure 5:**
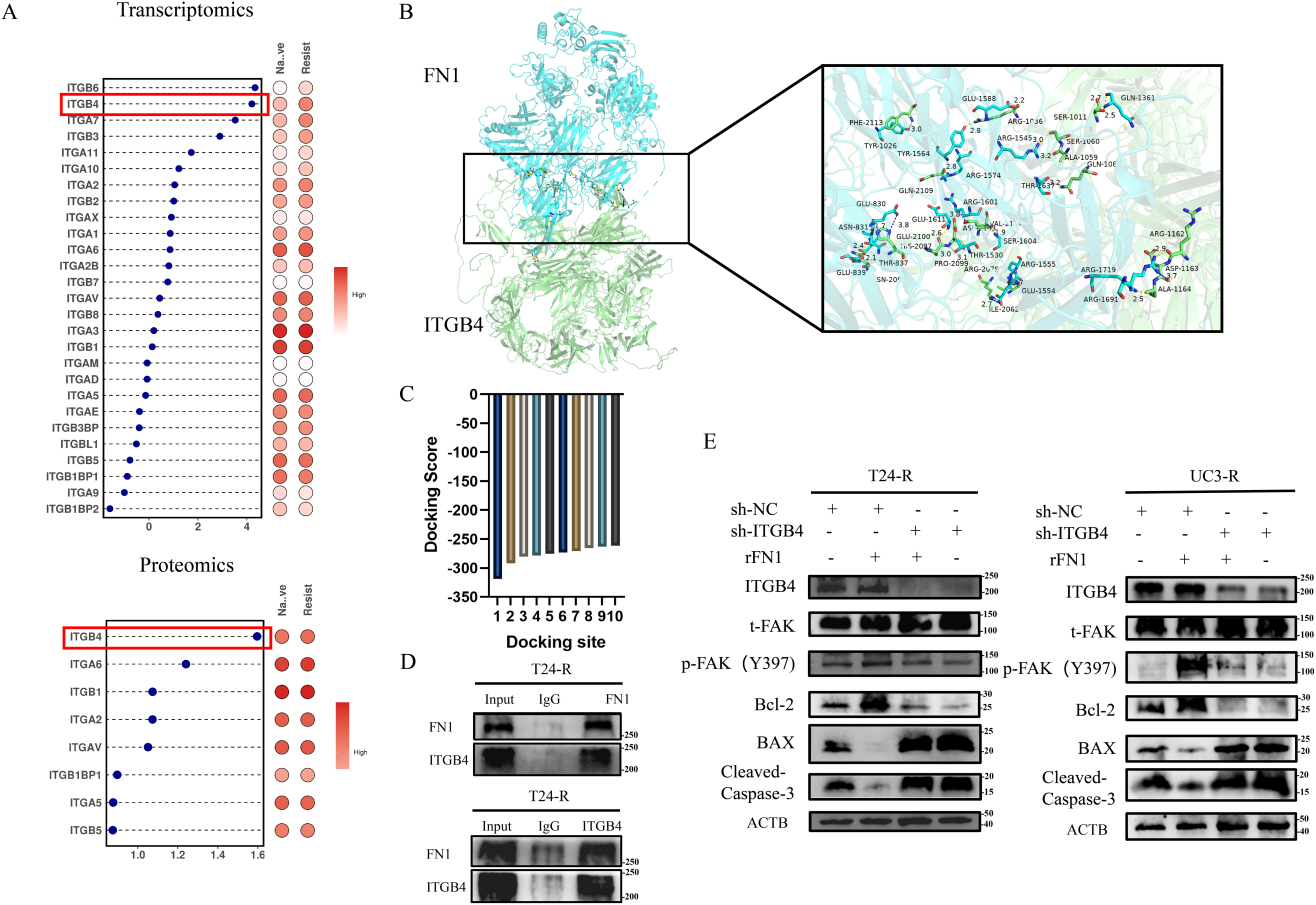
ITGB4 is critical for FN1-mediated chemotherapy resistance in bladder cancer cells. (**A**) Differential expression analysis revealed that ITGB4 (highlighted by the red box) was significantly overexpressed in T24-R cells compared to T24, suggesting a role in FN1-mediated resistance. (**B**) Structural modeling shows multiple binding sites between FN1 (blue ribbon) and ITGB4 (green ribbon). (**C**) The interaction between FN1 and ITGB4 had a binding score of −318.75 with a confidence score of 96%, indicating a stable interaction. (**D**) The Co-IP experiment confirmed that there is a mutual binding interaction between FN1 and ITGB4. (**E**) Adding rFN1 to resistant strains increased FAK (Y397) phosphorylation and inhibited apoptosis, whereas ITGB4 silencing reversed these effects, highlighting the dependency of FN1-mediated resistance on ITGB4 expression and activation. For panels (**A**,**C**,**E**), a *t*-test was used for comparisons between two groups, and one-way ANOVA was used for comparisons among multiple groups

By conducting a differential analysis of the highly expressed integrin family subunits in the transcriptomic and proteomic studies of T24-R and T24 (Fig. 5A), we found that ITGB4 was overexpressed in the resistant strains, suggesting that ITGB4 plays a critical role in FN1-mediated chemotherapy resistance. As shown in Fig. 5B, the blue ribbon structure represents FN1, and the green ribbon structure represents the ITGB4 protein, indicating multiple binding sites between FN1 and ITGB4. The binding score between FN1 and ITGB4 proteins was −318.75, with a confidence score of 96%, indicating a stable interaction between FN1 and ITGB4 (Fig. 5C). Subsequently, we have experimentally validated the interaction between FN1 and ITGB4 (Fig. 5D). Adding rFN1 to the resistant strains increases the phosphorylation levels of FAK (Y397) and inhibited apoptosis; however, silencing ITGB4 in the resistant strains reversed this effect (Fig. 5E). Therefore, we speculate that FN1-mediated chemotherapy resistance in bladder cancer cells depends on the expression and activation of ITGB4.

## 4 Discussion

Bladder cancer is one of the most common malignant tumors in urology, and cisplatin-based chemotherapy is one of its primary treatment methods [28,29]. However, chemotherapy resistance is a common issue in bladder cancer treatment, often leading to poor therapeutic outcomes. This study demonstrates elevated FN1 expression in both chemoresistant bladder carcinoma cell lines and clinical specimens. Furthermore, FN1 promotes chemoresistance in this malignancy by activating the ITGB4-FAK signaling axis.

FN1, as an extracellular matrix protein, can be secreted by cancer cells into the microenvironment. Content of FN1 has an impact on matrix stiffness and influences cellular motility via activating Rho-ROCK signaling through increasing cytoskeletal tension. Besides, up-regulation of FN1 is a marker of EMT in several tumors. FN1 is a high-molecular-weight glycoprotein composed of two subunits, containing multiple structural domains that enable it to perform various functions through binding with different receptors, such as integrin family located in cell membranes [30]. Our research found that knocking down FN1 can enhance the chemotherapy sensitivity of bladder tumor cell lines. Consequently, the activity of the FAK signaling pathway was significantly attenuated, as evidenced by reduced phosphorylation levels of FAK (Y397).

As one of the most important membrane receptors, integrin family plays a key role in heterotypic adhesion and chemotactic movement in several types of cells, such as tumor and immune cells. Integrins are a family of heterodimeric transmembrane receptors formed by non-covalent association of α and β subunits. Integrins in tumors typically function through mechanotransduction, stem cell regulation, epithelial plasticity, and therapy resistance. In breast cancer, Saatci et al. [6] found that downregulation of FN1 expression could inhibit the ITGA5-FAK-Src signaling pathway, thereby inducing apoptosis and restoring chemosensitivity in breast cancer cells. Similarly, Assidicky et al. [11] identified FN1 as a central resistance-driving gene. Targeting the FN1 receptor ITGA5 can reverse FN1-driven drug resistance. In gastric cancer, Gao et al. [31] discovered that FN1 directly interacts with integrin β1 protein in resistant cells. Our discovery of ITGB4 as the key partner in bladder cancer, as opposed to ITGA5 in breast cancer or integrin β1 in gastric cancer, suggests a tissue-specific mechanism of FN1-mediated resistance. This specificity highlights the importance of developing context-dependent therapeutic strategies targeting the FN1-integrin axis. FN1 silencing suppressed the Wnt/β-catenin signaling pathway, an effect that was inhibited by integrin β1 blocking antibodies. However, the mechanism by which FN1 mediates drug resistance in bladder cancer requires in-depth investigation. Our study revealed that FN1 mediates chemoresistance by inhibiting apoptosis through the ITGB4-FAK pathway, and that blocking ITGB4 can reverse FN1-driven chemoresistance. Our data, showing that genetic disruption of FN1 or ITGB4 restores cisplatin sensitivity both *in vitro* and *in vivo* (as evidenced by the xenograft model), strongly suggest that pharmacological inhibition of the FN1-ITGB4 interaction or of FAK kinase activity could be a viable therapeutic strategy to overcome cisplatin resistance in bladder cancer.

Despite these findings, our study has certain limitations that should be acknowledged. The sample size for the immunohistochemical validation in patient tissues was relatively small, which may limit the generalizability of our conclusions. Future studies involving larger, independent patient cohorts are necessary to confirm the clinical relevance and prognostic value of FN1 and ITGB4 in bladder cancer chemoresistance. Additionally, exploring the potential crosstalk between the FN1-ITGB4-FAK axis and other resistance mechanisms, such as enhanced DNA damage repair or drug efflux pumps, would provide a more comprehensive understanding of the chemoresistance landscape.

## 5 Conclusion

In conclusion, our study identifies the FN1-ITGB4-FAK signaling axis as a critical driver of acquired cisplatin resistance in bladder urothelial carcinoma. We demonstrate that targeting this pathway, either genetically or pharmacologically, can re-sensitize resistant cells to apoptosis and inhibit tumor growth *in vivo*. These findings nominate the FN1-ITGB4 interaction and FAK signaling as promising therapeutic targets to overcome chemotherapy resistance in bladder cancer patients.

## Supplementary Materials



## Data Availability

The datasets generated during and/or analyzed during the current study are available in the following repositories: the transcriptomic datasets are available in the NIH GEO repository under accession numbers GSE309386 (T24-R vs. T24) and GSE309388 (UC3-R vs. UC3); the proteomic dataset is available in the ProteomeXchange Consortium repository under accession number PXD069140. All other data are available from the corresponding author [Weiyang He], upon reasonable request.

## Supplementary Materials

The supplementary material is available online at https://www.techscience.com/doi/10.32604/or.2025.072084/s1.
