# Supplementary material for: The FN1-ITGB4 Axis Drives Acquired Chemoresistance in Bladder Cancer by Activating FAK Signaling
Source: Oncol Res. 2026 Jan 19;34(2):21. doi: 10.32604/or.2025.072084 (PMC12848685; doi:10.32604/or.2025.072084)

**Supplementary Figure S1.** Volcano plots of the omics data. Transcriptomic analysis of T24-R vs T24 (GEO: GSE309386). Transcriptomic analysis of UC3-R vs UC3 (GEO: GSE309388).

**
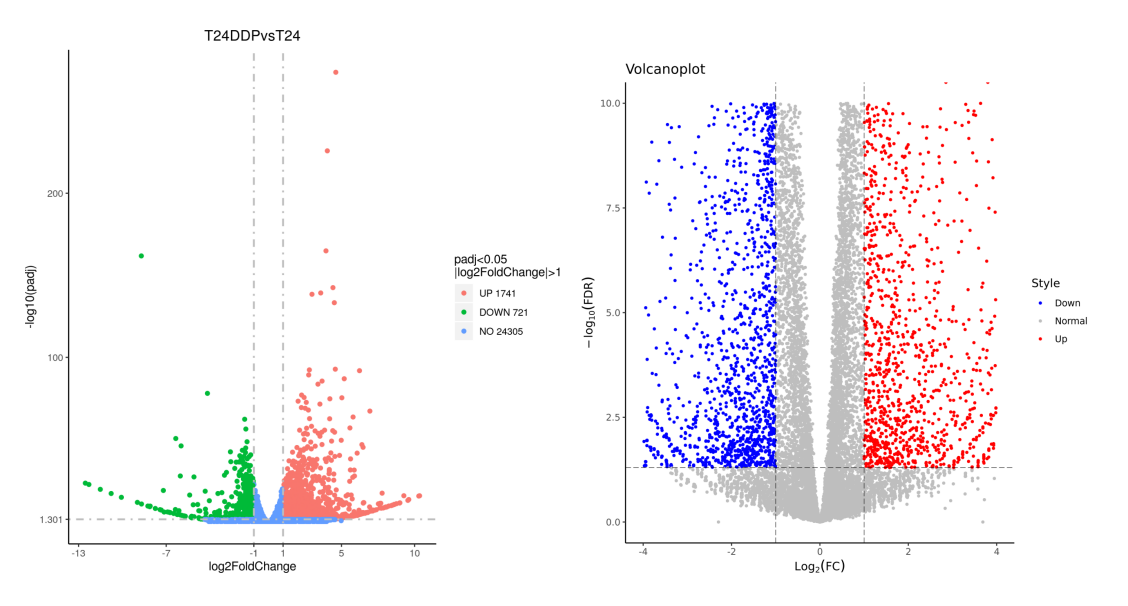
**

**Supplementary Figure S2.** In the drug-resistant cell lines, the knockdown of FN1 does not impact cellular apoptosis in the absence of cisplatin.

**
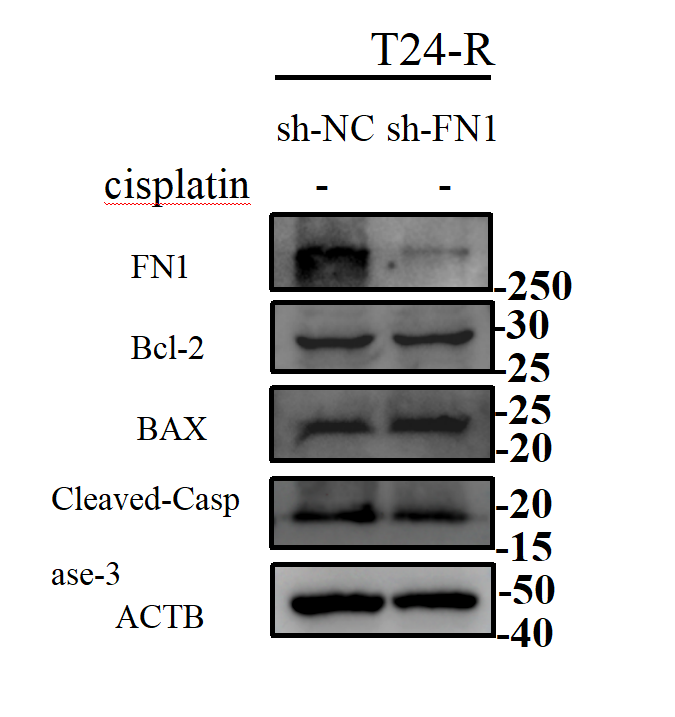
**

**Supplementary Figure S3.** The supernatants of the GC-resistant cell lines and GC-sensitive cell lines were collected after 48 hours of culture to detect the FN1 levels. It was found that the FN1 content in the supernatant of the GC-resistant cell lines was significantly increased. **p<0.01.

**
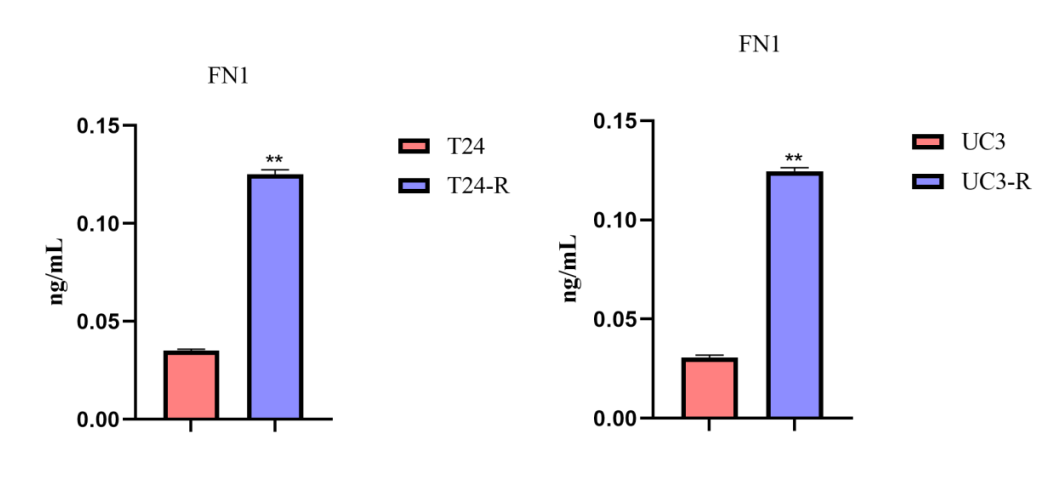
**

**Supplementary Figure S4.** The incorporation of rFN1 into T24-R has been shown to augment the phosphorylation of FAK at Y397. **p<0.01.


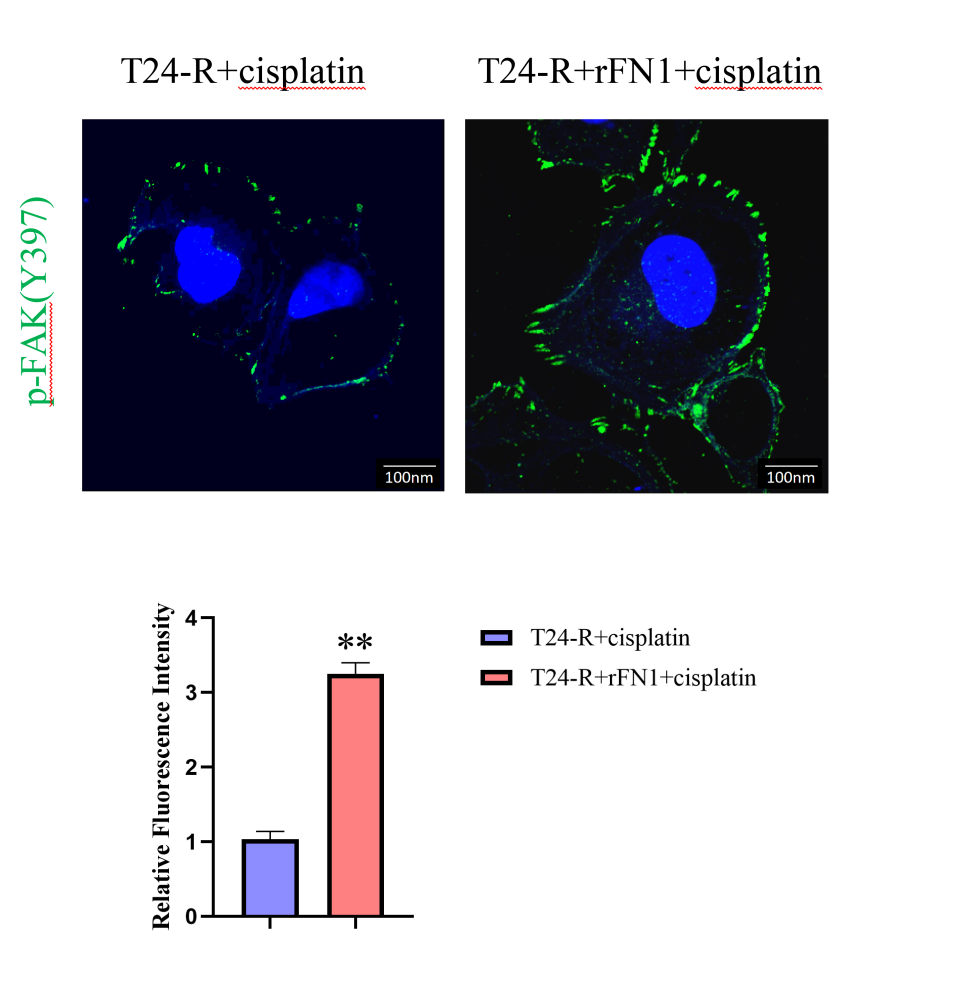

Supplement: Supplementary file 1 [file OncolRes-34-72084-s001.docx]
